# Supplementary material for: Time-series transcriptome analysis identified differentially expressed genes in broiler chicken infected with mixed Eimeria species
Source: Front Genet. 2022 Aug 8;13:886781. doi: 10.3389/fgene.2022.886781 (PMC9393255; doi:10.3389/fgene.2022.886781)
Supplement: Supplementary file 2 [file DataSheet1.ZIP › 7dpi_GO.Gsea.1625106495923/GOMF_PHOSPHORUS_OXYGEN_LYASE_ACTIVITY.html]

Details for gene set GOMF\_PHOSPHORUS\_OXYGEN\_LYASE\_ACTIVITY[GSEA]

|  || Dataset | TMM\_7dpi\_gct\_format\_7dpi\_gct\_format.Class\_4dpi.cls #PC\_versus\_NC.Class\_4dpi.cls #PC\_versus\_NC\_repos |
| Phenotype | Class\_4dpi.cls#PC\_versus\_NC\_repos |
| Upregulated in class | 0 |
| GeneSet | GOMF\_PHOSPHORUS\_OXYGEN\_LYASE\_ACTIVITY |
| Enrichment Score (ES) | -0.73913646 |
| Normalized Enrichment Score (NES) | -1.8484548 |
| Nominal p-value | 0.0 |
| FDR q-value | 0.098535925 |
| FWER p-Value | 0.763 |
Table: GSEA Results Summary

  

Fig 1: Enrichment plot: GOMF\_PHOSPHORUS\_OXYGEN\_LYASE\_ACTIVITY      
 Profile of the Running ES Score & Positions of GeneSet Members on the Rank Ordered List

  

| SYMBOL | TITLE | RANK IN GENE LIST | RANK METRIC SCORE | RUNNING ES | CORE ENRICHMENT || 1 | ADCY9 | na | 2142 | 0.246 | -0.1475 | No |
| 2 | NPR2 | na | 3645 | 0.048 | -0.2667 | No |
| 3 | TKFC | na | 5326 | -0.133 | -0.3899 | No |
| 4 | ADCY3 | na | 5416 | -0.141 | -0.3796 | No |
| 5 | ADCY6 | na | 5876 | -0.188 | -0.3940 | No |
| 6 | CD38 | na | 6007 | -0.202 | -0.3793 | No |
| 7 | GUCY1A2 | na | 10325 | -0.775 | -0.6412 | Yes |
| 8 | BST1 | na | 10551 | -0.820 | -0.5564 | Yes |
| 9 | ADCY8 | na | 10620 | -0.838 | -0.4563 | Yes |
| 10 | GUCY2C | na | 10748 | -0.866 | -0.3575 | Yes |
| 11 | ADCY2 | na | 11469 | -1.105 | -0.2780 | Yes |
| 12 | MOCS1 | na | 11730 | -1.269 | -0.1394 | Yes |
| 13 | ADCY7 | na | 11750 | -1.288 | 0.0217 | Yes |
Table: GSEA details [plain text format]

  

Fig 2: GOMF\_PHOSPHORUS\_OXYGEN\_LYASE\_ACTIVITY      
 Blue-Pink O' Gram in the Space of the Analyzed GeneSet

  

Fig 3: GOMF\_PHOSPHORUS\_OXYGEN\_LYASE\_ACTIVITY: Random ES distribution      
 Gene set null distribution of ES for **GOMF\_PHOSPHORUS\_OXYGEN\_LYASE\_ACTIVITY**
